# Receptor Tyrosine Kinases — Expanding Horizons

**DOI:** 10.3390/cells3020657

**Published:** 2014-06-20

**Authors:** Sassan Hafizi

**Affiliations:** School of Pharmacy and Biomedical Sciences, Division of Pharmacology, University of Portsmouth, UK; E-Mail: sassan.hafizi@port.ac.uk

This Special Issue of *Cells* on receptor tyrosine kinases (RTKs) is a timely and unique assemblage of scholarly insights into topics that have relatively recently entered the spotlight in relation to this class of molecules. The review by Julien *et al.* [1] is an overview of the knowledge on how gangliosides, constituting certain membrane microdomains, may interact with and regulate RTK activation and downstream signalling. Similarly, the review by Banning *et al.* [2] focuses on the influence of another type of membrane microdomain, namely that containing flotillins, on regulation of RTK signalling and its relevance to cancer. Both of these reviews provide novel insights into mechanisms of transmembrane receptor signalling that rely on the constitution of the microdomains the RTKs reside in, and how their modification may affect receptor clustering, activation and translocation. Thus, knowledge about such microdomains and their interactions with RTKs can provide new information on common regulation pathways starting at the membrane level, which could have implications for novel therapeutic angles in, e.g., cancer.

Although RTKs feature highly in cell proliferative and infiltrative diseases, such as cancer, it may be easy to forget that the presence and activity of RTKs is crucial to normal roles in cell and tissue development and homeostasis. The review by Brix *et al.* [3] highlights the importance of one such RTK, the EGFR subfamily member ErbB2, in maintenance of a normal physiological state, as well as how it works in a detrimental manner in disease, such as promoting cell survival, proliferation and invasion of breast cancer cells. An overview of RTKs and their ligands within the context of another cancer type, namely the brain tumour glioblastoma multiforme, is provided by Carrasco-Garcia *et al.* [4]. In this review, the role of RTKs in promoting this highly aggressive and difficult-to-treat CNS tumour is discussed, as well as the potential of targeting RTKs in therapy, as a means to extending the woefully short life expectancies these patients have.

The paradigm of ligand-induced receptor dimerisation as a model for RTK signalling initiation becomes less rigid when reading the review by Maruyama [5]. In it, the author draws upon results of studies that indicate alternative temporal sequences and structural models for RTK monomer interactions. These alternative models of dimerisation open up the possibilities for RTK signalling to occur perhaps through RTK heterodimerisation and/or activation by alternative ligands. Away from the cell periphery, the processes regulating RTK activation, signal transduction and sorting and recycling inside the cell are also topics of great interest that touch on other, distinct, aspects of RTK signalling. The article by Jopling *et al.* [6] reports on the role of Rab GTPases in endosome-to-plasma membrane recycling of an RTK, in this case VEGFR2, and how control of its trafficking is important to angiogenesis. Similarly, control of RTK intracellular trafficking is likely to be important for the strength and persistence of RTK signalling in other scenarios, such as cancer. An intracellular mediator of RTK signalling, the kinase Src, is the focus of the review by Mezquita *et al.* [7]. Here, they review observations on intracellular “fragments” of RTKs, such as KIT and VEGFR1, that seem to be regulated not by ligand binding at the plasma membrane, but by distinct signalling cues upstream, and are therefore able to activate Src in a discrete manner. This touches upon an interesting and burgeoning area of research, namely that focusing on the intracellular, and particularly nuclear, functions of RTKs, which is sure to provide significant insights into the novel potential of RTKs as direct gene regulatory molecules.

A further distinct sub-realm of cellular signalling is represented by the article by Goltsov *et al.* [8], who carried out a systems analysis of the effect of anti-RTK agents on reprogramming of RTK expression and signalling in breast cancer cells. This study is a good example of the application of the latest technologies and vast computing power available today for analysing multiple parameters in signalling networks. This powerful approach is sure to become ever more significant in helping to make sense out of the complexity and diversity of signal transduction networks, and may be especially useful in relation to individualised therapy in the future.
